# Hospital costs fell as numbers of LVADs were increasing: experiences from Oslo University Hospital

**DOI:** 10.1186/1749-8090-7-76

**Published:** 2012-08-27

**Authors:** Vinod Mishra, Arnt E Fiane, Odd Geiran, Gro Sørensen, Ishtiaq Khushi, Terje P Hagen

**Affiliations:** 1 Department of Finance and Resource Management Unit, Oslo University Hospital, Oslo, Norway; 2 Department of Cardiothoracic Surgery, Oslo University Hospital, Oslo, Norway; 3 Faculty of Medicine, University of Oslo, Oslo, Norway; 4 Health Services Research Center Akershus University Hospital, Oslo, Norway; 5 Department of Health Management and Health Economics, University of Oslo, Oslo, Norway

**Keywords:** Innovative technology, Left ventricle assist device, Hospital cost, DRG reimbursement, Cost analysis

## Abstract

**Background:**

The current study was undertaken to examine total hospital costs per patient of a consecutive implantation series of two 3^rd^ generation Left Ventricle Assist Devices (LVAD). Further we analyzed if increased clinical experience would reduce total hospital costs and the gap between costs and the diagnosis related grouped (DRG)-reimbursement.

**Method:**

Cost data of 20 LVAD implantations (VentrAssist™) from 2005-2009 (period 1) were analyzed together with costs from nine patients using another LVAD (HeartWare™) from 2009-June 2011 (period 2). For each patient, total costs were calculated for three phases - the pre-LVAD implantation phase, the LVAD implantation phase and the post LVAD implant phase. Patient specific costs were obtained prospectively from patient records and included personnel resources, medication, blood products, blood chemistry and microbiology, imaging and procedure costs including operating room costs. Overhead costs were registered retrospectively and allocated to the specific patient by predefined allocation keys. Finally, patient specific costs and overhead costs were aggregated into total hospital costs for each patient. All costs were calculated in 2011-prices. We used regression analyses to analyze cost variations over time and between the different devices.

**Results:**

The average total hospital cost per patient for the pre-LVAD, LVAD and post-LVAD for period 1 was $ 585, 513 (range 132, 640- 1 247, 299), and the corresponding DRG- reimbursement (2009) was $ 143, 192 . The mean LOS was 54 days (range 12- 127). For period 2 the total hospital cost per patient was $ 413, 185 (range 314, 540- 622, 664) and the corresponding DRG- reimbursement (2010) was $ 136, 963. The mean LOS was 49 days (range 31- 93).

The estimates from the regression analysis showed that the total hospital costs, excluding device costs, per patient were falling as the number of treated patients increased. The estimate from the trend variable was -14, 096 US$ (CI -3, 842 to -24, 349, p < 0.01).

**Conclusion:**

There were significant reductions in total hospital costs per patient as the numbers of patients were increasing. This can possibly be explained by a learning effect including better logistics, selection and management of patients.

## Background

Prevalence of heart failure has been increasing over the last decade and left ventricular assist device (LVAD) therapy has to an increasing degree become an option for patients in end-stage heart failure and also as an alternative to heart transplant (Htx). There are currently a variety of LVAD systems available, with different technology [1-4].

The aim of this study was to investigate how total hospital costs per patient developed as the number of LVAD procedures increased and clinical experience was accumulated. The data consisted of information of patients from two subsequent periods where two different 3^rd^ generation LVADs were used; in period 1 VentrAssist™LVAD (removed from the market in 2008) and in period 2 HeartWare™ LVAD (Framingham, MA, USA). Costs of the pre-LVAD work-up and treatment, the LVAD implantation phase as well as the post-LVAD care were evaluated in a setting of implementation of a new treatment program.

## Methods

### Patient material and treatment procedure

We have earlier published a cost analysis with data from our first nine VentrAssist™ LVAD implantations [5]. In this paper we expand the analysis to include 11 further implantations with VentrAssist™ LVAD from 2005-2009 (period 1) and nine consecutive implantations with HeartWare™ LVADs from 2009 to June 2011 (period 2). The cohort was recruited from patients evaluated for Htx. Two patients receiving biventricular implants were excluded from this analysis.

The normal pathway for LVAD patients was: 1) A pre-LVAD period where the patients were admitted to the Medical intensive care unit (MICU) for clinical evaluation, investigation per protocol and treatment. Seven patients were on Extracorporeal Membrane Oxygenation (ECMO), while most of the patients were on intra-aortic balloon pump (IABP) prior to the LVAD implantation. 2) A LVAD-phase including LVAD- surgical implantation, stay in the Thorax intensive care unit (TICU) and general ward. 3) The late post-LVAD course which started at the date of discharge and went on to either one year after implantation, date of Htx (if Htx was within one year after discharge) or death within the first year after implantation.

### Cost analysis

The analysis involved two sets of data, one based on data from the individual patient (direct costs) and one based on overhead costs (indirect costs), with the overhead costs ultimately also allocated to the individual patient. The basic principle of the analysis was to relate as much as possible of the resources used as direct costs to the individual patient. A detailed description of costing method is described in the previous article [5].

To compare cost between years all cost data were calculated in 2011-prices using the price index for public service production as deflator. We converted Norwegian Kroner (NOK) to US dollars with the exchange rate of 1 US $ = 5.80 NOK.

### Statistical methods

We evaluated trends in total hospital costs per patients and total hospital costs, excluding device costs, per patient by the means of multivariate regression analyses. Initial testing indicated that the best model fit was achieved by linear regression models. Two different specifications were estimated for each of the two dependent variables. Due to few degrees of freedom (few cases) the number of independent variables will be strongly limited. Both specifications included number of patients and days on ECMO. Number of patients worked as a trend variable and was simply described as a variable taking the value of 1 for the first LVAD-procedure and increasing by 1 for each consecutive treatment. Days on ECMO was entered to control for high risk patients. Additionally, we included a variable describing type of LVAD in one of the specifications. Type of LVAD was described by a dummy variable HeartWare™= 1 (Additional file 1: Appendix A). We considered P-values <0.05 as statistically significant and used SPSS 8 for all statistical analyses.

### DRG reimbursement

The actual DRG code for each patient is automatically created by a combination of ICD-10 diagnosis and procedure code based on the Nordic Classification of Surgical Procedures (NOMESCO) [6,7]. In Norway, no specific DRG for LVAD implantation existed at the time of this study. So far all patients treated with extra-, para- or intra-corporeal blood pumps (excluding IABP) had been classified as a cardiac implantation with a mechanical device and grouped with same DRG as Htx (DRG103). “Htx” had in 2009 a unit price of $ 143, 192 and in 2010 $ 136, 963. If the patient had both LVAD and Htx during the same hospital admission, only one DRG 103 was assigned. There are no revenues coming from out-of-pocket payment as in-patient stays are free of charge in Norway [8].

## Results

Among the 29 patients, 26 were men and three were female. Average age was 41 years (range 10- 72) at the time of inclusion. At the termination of our project 22 patients had received Htx, including one that had Htx during the LVAD-stay, but who died after the Htx-procedure. Four patients died without Htx, and three were still on device as outpatients. The mean length of hospital stay (LOS) for the three phases was 52 days (range 12-127), of which 18 days (range 5-56) was in the ICU (Table 1).

**Table 1:** Patients characteristics, LOS and endpoints

			**Pre-LVAD phase days)**		**LVAD phase (days)**	**Total in-hospital stay (days)**		**End point**
	**Patient #**	**Age (years)**	**Sex**	**LOS**	**ECMO LOS**	**LOS**	**LOS ICU**		**LOS**
VA 1	64	M	20		22	25	42	Htx
VA 2	62	M	1		26	7	27	†
VA 3	15	F	8		26	6	34	Htx
VA 4	38	M	5	6	28	27	33	Htx
VA 5	23	M	8		32	10	40	Htx
VA 6	54	M	1	14	26	18	27	Htx †
VA 7	65	M	8		29	11	37	*
VA 8	16	F	15	23	42	13	57	*
VA 9	20	M	13		21	5	34	Htx
VA 10	59	M	14		88	35	102	†
VA 11	55	M	8		39	39	47	†
VA 12	53	F	12		32	21	44	Htx
VA 13	10	M	6		39	14	45	Htx
VA 14	19	M	49		42	17	91	Htx
VA 15	27	M	39	11	56	18	95	Htx
VA 16	29	M	2		125	56	127	Htx
VA 17	32	M	32		45	23	77	Htx
VA 18	21	M	44		25	14	69	Htx
VA 19	61	M	1		40	12	41	Htx
VA 20	16	M	8	4	4	12	12	Htx
HW 1	39	M	0	3	41	15	41	Htx
HW 2	72	M	5		42	21	47	Htx
HW 3	56	M	29		29	8	58	*
HW 4	62	F	11		31	12	42	†
HW 5	54	M	36		57	40	93	Htx
HW 7	61	M	6	7	27	9	33	Htx
HW 8	26	M	12		19	7	31	Htx
HW 10	35	M	17		40	6	57	Htx
HW 11	46	M	6		30	13	36	Htx
Mean	41		14	10	38	18	52	
Range	(10-72)		(1-49)	(3-23)	(4-125)	(5-56)	(12-127)	

*VA* VentrAssist™LVAD.

*HW* HeartWare™LVAD.

*M* Male.

*F* Female.

*LOS* (Length of Stay) in days.

*ECMO* Extra Corporeal Membrane Oxygenation.

*ICU* Intensive Care Unit.

*Htx* Heart transplantation.

End points: † = dead, * = on device.

^^ patient no 6 and 9 from the HeartWare LVAD study was excluded as patient had HW BIVAD.

**Mean cost for ECMO is only for the patients with ECMO.

**Figure 1:**
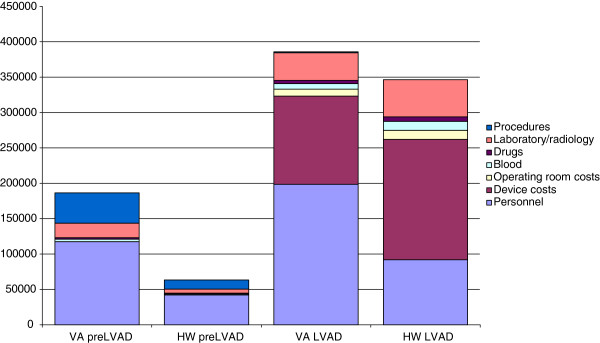
**Costs ($) per patient per LVAD phase and cost drivers for 20 VentrAssist**™ **LVAD and 9 HW HeartWare**™ **LVAD.**

Disaggregating the total costs for first 20 patients using VentrAssist™LVADs into the different phases revealed that the mean costs for the pre-LVAD phase were $ 186, 467 (range 6, 000- 586, 421) (Table 2). The major cost drivers in this phase were personnel cost (63%) and ECMO-procedure cost (23%) (Figure 1). The mean LOS in the pre-LVAD phase was 15 days (range 1- 49). In the LVAD-phase mean costs were $ 378, 450 (range 41, 957- 696, 483). The major cost drivers in this phase were personnel costs (52%) and device costs (32%). The mean LOS for the LVAD phase was 39 days (range 4- 125).

**Table 2:** **Average patient costs ($) per LVAD phase, cost drivers per phase and average total costs ($) for 20 VentrAssist**™ **LVAD and 9 HW HeartWare**™**LVAD**

		***Total costs in $ (range)***
**20 VentrAssist ™ LVAD**		**9 HeartWare™LVADs**
Pre-LVAD phase	**186, 467 (6, 000- 586, 421)**	**63, 963 (9,738- 187, 097)**
* Cost drivers %*		
Personnel	63	*43*
ECMO procedure	23	*42*
Blood	9	*6*
Drugs	1	*1*
Laboratory/radiology	4	*8*
* Sum*	*100*	*100*
LVAD phase	**378, 450 (41, 957- 696, 483)**	***346, 403 (273, 498- 425, 843)***
* Cost drivers %*		
Personnel	52	*27*
Device costs	32	*58*
Operating room cost	11	*10*
Blood	1	*2*
Drugs	1	*1*
Laboratory/radiology	3	*2*
* Sum*	*100*	*100*
Post- LVAD phase	**18,093 (8,729,- 59, 345)**	***2, 819 (1,310- 9,724)***
* Cost drivers %*		
Day hospital stays (under 5 hours)	69	*0*
In-hospital stays	27	*55*
External consultations	2	*22*
Internal consultations	2	*23*
* Sum*	*100*	*100*
Mean total costs	**585, 513**	***413 185***
Range	**(132, 640- 1 247, 299**)	**(314, 540- 622, 664)**

For the subsequent nine patients using the new device (HeartWare™) the mean costs for the pre-LVAD phase were $ 63, 963 (range 9, 738- 187, 097). The major cost drivers in this phase were personnel cost (43%) and ECMO-procedure cost (42%). Two patients had three and seven days on ECMO before the LVAD implantation. Five patients were on IABP in Medical intensive care unit (MICU). The mean LOS in the pre-LVAD phase was 14 days (range 0- 36). In the LVAD-phase mean costs were $ 346, 403 (range 273, 498- 425, 843). The major cost drivers were device costs (58%) and personnel cost (27%). The mean LOS for the LVAD phase was 35 days (range 19- 57). In the post-LVAD phase mean costs were $ 2, 819 (range 1, 310- 9, 724). Device costs were $ 121,280 for VentrAssist™ LVAD and $ 200,913 for HeartWare™.

**Figure 2:**
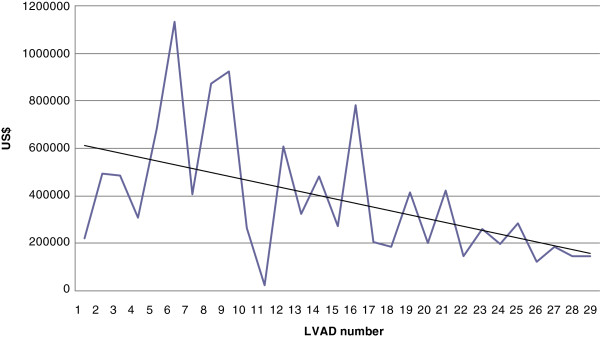
Correlation between total cost excluding device costs and number of LVAD implantations.

Of the two different specifications the ones without a dummy variable describing type of LVAD perform best for both dependent variables. Of the analyses of the two dependent variables the one where device costs are excluded have the best performance (Adjusted r R^2^). The estimates from the regression analysis of total costs excluding device costs indicated that costs were falling as the number of treated patients increased (Table 2, Figure 2). The estimate from the trend variable was - $ 14,096 (CI -3,842 to -24,349, p < 0.01) indicating that for each additional patient, total LVAD-costs excluding device costs, were reduced on average.

A number of adverse effects were observed (Table 3). The most common device related adverse effects within 30 days were four systemic or deep infections and one patient had percutaneous lead exit site infection. None of the patients had device malfunction necessitating removal or change of parts of the systems.

**Table 3:** Adverse events

***Adverse events***	***≤30 days events***		***>30 days events***	
	**VA**	**HW**	**VA**	**HW**
Local Infection	1	1	3	2
*Percutaneous lead exit site*			*1*	*4*
*Pump pocket*	*1*		*2*	*0*
Systemic (sepsis)	1	0		
Neurological event	3	2	2	0
*Hemorrhagic*	*1*	*1*		
*Embolic*	*2*	*1*	*2*	*0*
* Resolved (no disability)*			*2*	
* Minor disability*	*3*			
Bleeding in need of surgery	1		0	0
System malfunction affecting therapy	0	0	0	0

## Discussion

There have been attempts to analyze true hospital cost of LVAD [9-11]. However to our knowledge there are no studies where costs of two different devices have been in dept analyzed. In this study we have explored the effect of accumulated clinical experience. We have documented costs related to the LVAD procedure which is a necessary step to evaluate the procedure’s cost efficiency. Further, this analysis focuses on the existing reimbursement issues.

The mean total cost for the LVAD procedure is largely driven by the pre-LVAD phase where significant personnel resources were used commonly related to ICU treatments. In the LVAD phase the major part of cost was related to device cost. However, as different LVADs systems in pipeline are offered by different manufacturing companies and indications will be expanded to a more widespread use, it is likely that device costs will be reduced in the future.

It is worthwhile to mention that total cost for all three phases was almost 30% lower (from $ 585, 513 to $ 413, 185) in the second era using the new device for intrapericardial placement (HeartWare™LVAD) with even higher device cost compare to (VentrAssist™LVAD). On the other hand in first period most instances patients also required more extensive surgery. The total mean cost for pre LVAD phase of the first 20 VentrAssist™LVADs to nine HeartWare™LVADs the total mean cost for HeartWare™LVAD was 62% lower (186, 467 to 63, 963), indicating a more aggressive attitude to LVAD implantation than in the first study period.

The unit costs of personnel resources used in LVAD phase with the first 20 patients were 20% higher than in the second period, while the device cost for HeartWare™LVAD was 26% higher compared to VentrAssist™LVAD. Thus, for the LVAD phase taken together the reduction in total costs was only 9% (from $ 378, 450 to $ 346, 403). The total mean cost for post LVAD phase of the first 20 VentrAssist™LVADs to nine HeartWare™LVADs was considerably higher and can be explained by fact that first study period patients post LVAD phase check up was regularly at hospital. In second study period this function was allocated to local hospital.

The falling trend was also observed for the total in-hospital mean LOS. The total LOS for the pre LVAD and LVAD phase in the second period was lower than compared to the first period.

Although the results from the regression analyses should be regarded as indicative as the number of units are low, the gradually declining costs were confirmed. The marginal cost reduction was $ 14, 096 or 3.7% per patients calculated on the basis of total hospital costs excluding device costs. Within the range of this analysis there was no indication that the cost reductions were decreasing. The results should be considered in the light of the fact that by the first patients our institution was trying to establish a new treatment program. Costs at this stage were associated with low volume and high use of manpower. The higher cost among the first patients can be explained by longer LOS and more invasive treatment. These findings are also documented by other authors [12,13]. As the number of patients increased the department experienced learning effect from better logistics, selection and management of patients [14-17].

There are no studies that are exactly comparable in the literature. To our knowledge this is the first published study with an evaluation of the total patient cost including cost for pre-LVAD, LVAD and post LVAD period up to one year follow-up for two different consecutively used devices in a single institution program.

Innovative medical technology has impact on costs, but may with careful introduction improve health outcomes and quality of life substantially. First, when a technology/treatment becomes less expensive and safer fewer complications, meaningful improvements in quality of life, more patients may decide that a treatment is worth the risks and unpleasantness [18].

Efficacy and safety of LVAD have been reported in several studies [19,20]. However, there is no strong evidence that LVAD is a cost-effective choice of treatment for bridge to transplant patient group [21,22]. A recent study from Roger JG [23] at el indicate that LVADs appear beneficial, improve survival and quality of life but also that the cost per quality adjusted life year was high. Although LVADs may appear clinically effective as an alternative to HTx it is unlikely they will be cost-effective unless a significant cost decrease and a reduction in complications and a quality of life, will be proven. Further research in our institution should also address the cost effectiveness of the LVAD program in the long term as a bridge to Htx and as well for chronic LVAD patients.

Assuming that our cost estimates are correct, the discrepancy between cost and reimbursement illustrates one of the basic problems by the price setting in the DRG-system; costly innovations and implementations are not reflected in the prices of the system at early stages [24]. At best 3-4 years are spent to recalculate the cost weights of the system after an innovation is implemented. However, after our previous study documentation of cost and DRG reimbursement policy maker agreed upon that there was a need to allocate a specific DRG for LVADs in Norway. For 2012 (Activity based financing 2012) there will be a share of DRG reimbursement for LVADs [25]. However, this study also raises another discussion. Since standard reimbursement cannot cover the costs of the innovative phases in hospital treatments specific grants to innovation processes should be considered.

## Conclusions

The hospital costs per patients were falling as the numbers of LVADs were increasing. The higher cost among the first patients can be explained by longer LOS and more invasive treatment in the pre-LVAD phase. As the number of patients increased the department experienced learning effect from better logistics, selection and management of patients. This reduced total costs excluding device costs by approximately $ 14, 000 or 3.6% per each additionally treated patient.

## Abbreviations

LVAD: Left ventricular assist device; VA: VentrAssist™LVAD; HW: HeartWare™LVAD; LOS: (Length of Stay); ECMO: Extra Corporeal Membrane Oxygenation; ICU: Intensive Care Unit; Htx: Heart transplantation; MICU: Medical intensive care unit; TICU: Thorax intensive care unit.

## Competing interests

The author declares that they have no competing interests.

## Authors' contributions

All authors have read and approved the final manuscript. VM: Designed the study, conducted the study, analyzed the data, and wrote the manuscript. AF: wrote the manuscript. OG: wrote the manuscript. GS: wrote the manuscript. IK: analyzed the data. TPH : analyzed the data, wrote the manuscript. All authors read and approved the final manuscript.

## Supplementary Material

Additional file 1 **Appendix.** Regression analysis results. Click here for file
