# Supplementary material for: Hospital costs fell as numbers of LVADs were increasing: experiences from Oslo University Hospital
Source: J Cardiothorac Surg. 2012 Aug 27;7:76. doi: 10.1186/1749-8090-7-76 (PMC3515474; doi:10.1186/1749-8090-7-76)
Supplement: Additional file 1 — Appendix. Regression analysis results. [file 1749-8090-7-76-S1.doc]

Appendix A. Appendix

Regression analysis results

|  | **Hospital costs per patient** | | **Hospital costs, excluding device costs, per patient** | |
| --- | --- | --- | --- | --- |
| Variables | A | B | A | B |
| Intercept | 660854** | 647748** | 539574** | 554351** |
| Number of patients | -12230 | -10575* | -12229 | -14096** |
| Days on ECMO | 18300** | 18259* | 18301* | 18348* |
| Type of LVAD (HeartWare^TM^ = 1) | 37430 | - | -42203 |  |
| Adjusted R^2^ | .23 | .26 | 0.31 | 0.34 |

* = sig. < 0.05, ** = sig. < 0.01
